# Call for Specialty-Specific Benchmarks for Cross-Specialty Quality Measures in the Quality Payment Program

**DOI:** 10.7759/cureus.61618

**Published:** 2024-06-03

**Authors:** Kinan Sawar, Lana Sawar, Kevin Chen

**Affiliations:** 1 Plastic and Reconstructive Surgery, Wayne State University School of Medicine, Detroit, USA; 2 Internal Medicine, Southern Illinois University School of Medicine, Springfield, USA

**Keywords:** merit-based incentive payment system, performance evaluation, equity in healthcare, quality measures, medicare reimbursement, quality payment program

## Abstract

The introduction of the Quality Payment Program (QPP) by the Centers for Medicare & Medicaid Services (CMS) played a critical role in the process of transitioning U.S. healthcare from a pay-for-service to a pay-for-performance system. Physicians can participate in the QPP through one of three reporting methods: the traditional merit-based incentive payment system (MIPS), MIPS Value Pathways (MVPs), or Advanced Alternative Payment Models (APMs). These reporting methods require physicians to submit data on quality measures, which are averaged to determine a total quality performance score, which is weighted along with other QPP measures related to self-performance to provide an aggregate final performance score. This final score is used to determine either a negative, neutral, or positive percentage modifier for the physician’s Medicare reimbursement payments, which applies to the fiscal year two years following the year of reporting. Quality measures are either specialty-specific or cross-specialty, meaning that they are reportable by any physician specialty. No studies have compared performance across physician specialty categories on these measures. Critics argue that CMS has not ensured equitable reporting of cross-specialty quality measures due to the difference in emphasis on aspects of care of different physician specialties, potentially advantaging some. For example, family medicine physicians may score higher on the blood pressure control quality measure due to its relevance in their practice. Significant performance differences could highlight areas of improvement for certain physicians in certain specialties and guide balanced measure development. The QPP currently uses non-specialty-specific historical quality performance scores as benchmarks to determine current-year quality measure scores, likely leading to unfair comparisons. Establishing specialty-specific benchmarks for cross-specialty measures would promote equitable evaluation and fair competition among all participating physicians.

## Editorial

Introduction to the Quality Payment Program

Each year from 1998 to 2015, the Centers for Medicare & Medicaid Services (CMS) calculated physician reimbursement rates for physician services for patients utilizing Medicare using a formula called the Medicare Sustainable Growth Rate (SGR). The Medicare Access and CHIP Reauthorization Act of 2015 (MACRA) ended the use of this formula, and from 2016 to 2019, the reimbursement rates for Medicare were increased by 0.5% per year [1]. Beginning in 2019, the Medicare reimbursement rates for physician services began to be determined by the Quality Payment Program (QPP), which rewards physicians for high-value, high-quality care. This was a major step in transitioning U.S. healthcare away from a pay-for-service system toward a pay-for-performance system. There are three ways for physicians to participate in the QPP: either through the traditional MIPS (merit-based incentive payment system), MIPS Value Pathways (MVPs), or Advanced Alternative Payment Models (APMs) [2]. Within these participation methods, physicians are required to submit data on self-performance from a list of quality measures and consequently receive a score from 0 to 10 for each quality measure. These quality measure scores are then aggregated to achieve an average quality score, which is then weighted along with other QPP measures related to self-performance to provide an aggregate final score. This final score is used to determine either a negative, neutral, or positive percentage modifier for the physician’s Medicare reimbursement payments, which applies to the fiscal year two years following the year of reporting.

Some of the quality measures available for reporting are specialty-specific quality measures, while others are cross-specialty quality measures that are available for reporting by any physician specialty. The cross-specialty quality measures approved by CMS for the year 2024 include BMI screening and follow-up plan, documentation of an advance care plan, documentation of current medications in the medical record, tobacco use screening and cessation intervention, control of high blood pressure, and the patient activation measure (PAM), which assesses a patient’s ability to manage health [3]. No research study exists that has compared the performance of physicians of different specialties on these cross-specialty quality measures.

Criticism of cross-specialty quality measures

CMS believes cross-specialty measures significantly impact patient health outcomes, warranting their reporting by all physicians. However, CMS has failed to recognize the importance of ensuring that the cross-specialty quality scores are similar among physicians of all specialties each year so that physicians with particular specialties do not have a structural advantage in reporting these cross-specialty quality measures over other specialties. For example, family medicine physicians likely score higher than other specialties on the high blood pressure control quality measure due to its relevance in their practice. If significant differences in quality measure outcome scores between physicians of different specialties exist, then this data could inform areas of improvement for poorly performing specialties and may inform the future development of cross-specialty measures that will balance scores across all specialties.

The differential performance on these quality measures could be due to differing patient population characteristics and inherent differences in the perceived responsibility of physicians of differing specialties to optimize the quality of care related to these measures among other factors. Currently, the QPP uses historical average score performance in the most recent two years of reporting to create score benchmarks to evaluate how well a physician is performing on each quality measure. For example, a score of 5 corresponds to a percentile rank of 50th percentile relative to historical score performance with scores of 0 and 10 representing 0% and 100% percentiles, respectively. Implementing specialty-specific benchmarks could be easily done by stratifying historical performance data on these quality measures and finding benchmark values by primary physician specialty and calculating performance for these measures using these specialty-specific benchmarks. However, these benchmarks are used for all specialties. In other words, the benchmarks are not specialty-specific. Therefore, a case for specialty-specific benchmarks of the cross-specialty quality measures may be made if a significant difference in quality outcomes between physicians of different specialties is found. In fact, even if it is found that quality measure performance is not significantly different among specialties for the first eight years of QPP reporting (2017-2024), creating specialty-specific benchmarks for cross-specialty quality measures will ensure equitable comparison of all physicians participating in the QPP going forward. As the transition to quality-based healthcare in the United States continues, it will be crucial to ensure that healthcare policies are implemented in a way such that physicians are equitably being assessed and are rewarded for practicing value-based care rather than for optimizing quality of care reporting strictly for compensation gain.
